# Acquiring multiple slices in a single breath-hold. Is it practical for routine workflow?

**DOI:** 10.1186/1532-429X-11-S1-T3

**Published:** 2009-01-28

**Authors:** Ricardo Wage, John-Paul Carpenter, Dudley J Pennell

**Affiliations:** Royal Brompton and Harefield NHS Trust, London, UK

**Keywords:** Cardiovascular Magnetic Resonance, Ventricular Volume, Free Precession, Single Slice, Steady State Free Precession Sequence

## Introduction

The acquisition of the ventricular short axis cine stack forms the backbone of the routine cardiovascular magnetic resonance scan [1]. From this, the left and right ventricular volumes are calculated and important information is gained about wall thickness, regional function and evidence of dyssynchrony. Steady-state free precession cine (SSFP) loops can take between 8 and 12 seconds each to acquire and traditionally, a single breath-hold has been required for each slice. Parallel imaging technology has allowed reduction in the time taken for each acquisition without a significant drop in signal-to-noise ratio. As a consequence, it is possible to acquire two slices for each breath-hold. The scanner can be easily programmed to acquire a set of equally spaced ventricular short axis slices [2].

## Purpose

The purpose of this study was to assess the practicality of using a two-slice per breath-hold ventricular short axis cine sequence in routine daily practice.

## Methods

From the beginning of March 2008 to September 2008, we used a two-slice per breath-hold steady state free precession sequence to acquire the ventricular short axis stack of cines in a total of 478 patients. All patients were scanned with a 1.5 T Siemens scanner (Sonata or Avanto, Siemens, Erlangen, Germany) using anterior phased-array coils and ECG gating. Sequence parameters for the SSFP cine were as follows: 2 slices (8 mm slice thickness), 25% distance factor (2 mm gap), TR 40.2 ms, TE 1.13 ms, flip angle 80°, base resolution 192, number of signal averages 1, parallel imaging (GRAPPA; generalised autocalibrating partial parallel acquisition), bandwidth 930 Hx/Px, echo spacing 2.7 ms. Ten patients had both two-slice and single slice per breath-hold cine stack acquired. The parameters for the single slice acquisition were identical to those given above apart from using a 7 mm slice thickness with 3 mm gap.

## Results

**Figure 1:**
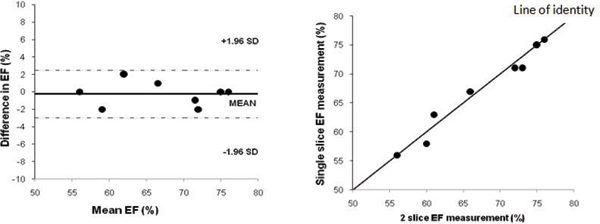
**Bland-Altman plot of difference in ejection fraction (EF) and scatter plot using 2-slice versus single slice acquisition**.

Since the beginning of the study, there have been no problems with the acquisition of the short axis stack using this technique. The images have all been suitable for analysis and calculation of ventricular volumes despite slightly increased partial volume effects towards the apex as a result of using an 8 mm slice thickness. In patients who had undergone both techniques, there was a low coefficient of variation between the two with no significant difference in volume or ventricular function calculations (see Figure 1 and Table 1).

**Table 1:** Coefficient of variation between scan techniques for single-slice and two-slice per breath-hold acquistion.

Parameter	Mean value	Mean difference between techniques (+/- in brackets)	Average coefficient of variation (%)	P value
LV end-diastolic volume (ml)	144.7	3.4 (6.1)	4.23	0.41
LV end-systolic volume (ml)	46.7	1.1 (3.72)	7.97	0.43
Stroke volume (ml)	97.8	2.4 (3.5)	3.58	0.43
LV mass (g)	133.3	2.2 (9.4)	7.04	0.45
Ejection fraction (%)	67.3	0.25 (1.39)	2.07	0.47

## Conclusion

In conclusion, the use of a two-slice per breath-hold cine acquisition is a practical method for use in daily practice. This shortens the time required for the whole ventricular short axis cine stack and allows a streamlining of workflow with overall reduction in the time taken for each CMR study. There is no significant difference in the ventricular volumes, mass or ejection fraction calculated.
